# Review of Journal of Cardiovascular Magnetic Resonance 2013

**DOI:** 10.1186/s12968-014-0100-2

**Published:** 2014-12-05

**Authors:** Dudley John Pennell, Arun John Baksi, Philip John Kilner, Raad Hashem Mohiaddin, Sanjay Kumar Prasad, Francisco Alpendurada, Sonya Vidya Babu-Narayan, Stefan Neubauer, David Nigel Firmin

**Affiliations:** Cardiovascular Biomedical Research Unit, Royal Brompton Hospital, Sydney Street, London, SW3 6NP UK; Imperial College, London, UK; University of Oxford, Oxford, UK

## Abstract

There were 109 articles published in the Journal of Cardiovascular Magnetic Resonance (JCMR) in 2013, which is a 21% increase on the 90 articles published in 2012. The quality of the submissions continues to increase. The editors are delighted to report that the 2012 JCMR Impact Factor (which is published in June 2013) has risen to 5.11, up from 4.44 for 2011 (as published in June 2012), a 15% increase and taking us through the 5 threshold for the first time. The 2012 impact factor means that the JCMR papers that were published in 2010 and 2011 were cited on average 5.11 times in 2012. The impact factor undergoes natural variation according to citation rates of papers in the 2 years following publication, and is significantly influenced by highly cited papers such as official reports. However, the progress of the journal's impact over the last 5 years has been impressive. Our acceptance rate is <25% and has been falling because the number of articles being submitted has been increasing. In accordance with Open-Access publishing, the JCMR articles go on-line as they are accepted with no collating of the articles into sections or special thematic issues. For this reason, the Editors have felt that it is useful once per calendar year to summarize the papers for the readership into broad areas of interest or theme, so that areas of interest can be reviewed in a single article in relation to each other and other recent JCMR articles. The papers are presented in broad themes and set in context with related literature and previously published JCMR papers to guide continuity of thought in the journal. We hope that you find the open-access system increases wider reading and citation of your papers, and that you will continue to send your quality manuscripts to JCMR for publication.

## Vessel Wall & MR Angiography

Beside its key role in research, CMR is most frequently used clinically for the carotid arteries where it can be used to characterize thrombus, [1] plaque vulnerability, [2] atheroma burden, natural history of progression, and response to treatment. [3] However, peripheral artery angiography, [4] and the role in investigation of pulmonary hypertension, [5-8] vasculitis and systemic hypertension, [9,10] is also making progress. This is an area of CMR where 3T has had significant impact [11].

### Vessel-wall imaging and quantification of flow-mediated dilation using water-selective 3D SSFP-echo

A new flow suppression method for vessel wall imaging using a steady state free precession (SSFP) sequence at 3T was described [12]. Flow sensitive water selective excitation SSFP echo that results in spoiling of signal in water protons of flowing blood due to phase incoherence was used. Flow mediated dilation (FMD) was also obtained from a peripheral artery. The proposed approach discriminates effectively between vessel wall and lumen. FMD results were qualitatively similar to values from literature. Although measurement of FMD is acquired from a very small group of individuals, it is still of reader interest and this technique may be more useful at higher field strengths.

### Steady state vascular imaging with extracellular gadobutrol: evaluation of the additional diagnostic benefit in patients who have undergone a peripheral magnetic resonance angiography protocol

This paper details the additional diagnostic benefits that can be achieved with imaging during the blood-pool phase following injection of an extracellular contrast agent for peripheral contrast-enhanced angiography [13]. Although the study is retrospective, the analysis, discussion and references list are comprehensive.

### Wall shear stress measured by phase contrast cardiovascular magnetic resonance in children and adolescents with pulmonary arterial hypertension

Phase-contrast imaging of the right pulmonary artery was used to quantify local, temporal and circumferentially averaged wall shear stress (WSS) and cross-sectional area of the right pulmonary artery over the cardiac cycle in children with PAH and young healthy adults [14]. In the presence of preserved flow rates through a large PAH pulmonary artery, WSS was shown to be significantly decreased. This may have implications for proximal pulmonary artery remodeling and cellular function in the progression of PAH.

### In-vivo quantitative T2 mapping of carotid arteries in atherosclerotic patients: segmentation and T2 measurement of plaque components

This manuscript describes the quantitative T2 mapping of carotid atherosclerotic plaques together with a semi-automated classification method that segments the T_2_ maps into four classes: calcification, lipid rich necrotic core, fibrous tissue and recent intraplaque haemorrhage [15]. The overall agreement between semi-automated classification of T2 maps compared to a gold-standard manual segmentation in terms of overall AHA plaque-type classification was good. The main limitations of the study are is the lack of histological confirmation, which the authors acknowledge.

### A case of a lesion containing an intracoronary thrombus detected as hyperintense plaque on T1-weighted cardiovascular magnetic resonance in a patient with silent myocardial ischemia

This case report concerns non-invasive visualisation of an intracoronary thrombus using T1 weighted inversion recovery MRI in a patient with stable angina [16]. This was subsequently confirmed by optical coherence tomography and X-ray angiography. Findings from Thallium-201 SPECT, coronary CTA and coronary MRA were also discussed.

### Comparison of symptomatic and asymptomatic atherosclerotic carotid plaques using parallel imaging and 3 T black-blood in vivo CMR

The authors implemented a rapid multi-contrast carotid plaque imaging protocol combining the increased SNR of a 3T MR scanner with rapid acquisition using parallel imaging [17]. The results demonstrate the advantage of the multi-contrast MR approach to characterize various carotid plaque components over just one MR sequence to detect carotid plaque haemorrhage. The reduced scanning time would be particularly useful for imaging sick patients with acute stroke. The authors demonstrated that symptomatic lesions can be identified but some of the parameters showed overlap between the symptomatic and asymptomatic lesions.

### SHILO, a novel dual imaging approach for simultaneous HI-/LOw temporal (Low-/Hi-spatial) resolution imaging for vascular dynamic contrast enhanced cardiovascular magnetic resonance: numerical simulations and feasibility in the carotid arteries

This manuscript describes a pulse sequence modification that allows low resolution images to determine arterial input function for pharmacokinetic modelling of contrast agent uptake in the carotid wall [18]. Only 5 patients were studied and there is no histological comparison of results. Further investigation of this technique in wider studies measuring kinetic parameters of plaque neovascularization and validation against gold standard techniques are required.

### Quantitative CMR markers of impaired vascular reactivity associated with age and peripheral artery disease

The authors present a comprehensive CMR study of vascular reactivity and vascular stiffness in patients with peripheral arterial disease (PAD) and healthy volunteers (young and old) [19]. Pre- and post-occlusive arterial hyperemia was determined in the femoral artery and vein by time-resolved blood flow velocity assessment and by the assessment of the haemoglobin oxygenation level. Vascular stiffness was determined by the pulse wave velocity, assessed in the aortic arch. Compared to volunteers, patients with PAD had a longer washout time of haemoglobin oxygen saturation, and a delayed and decreased response. Antegrade femoral arterial flow was prolonged, pulsatility was decreased and pulse wave velocity was increased. The proposed method represents an initial step towards development of a potential tool for evaluation of intervention.

### Reproducibility of rest and exercise stress contrast-enhanced calf perfusion magnetic resonance imaging in peripheral arterial disease

The inter-study and interobserver reproducibility as well as potential utility of calf stress perfusion was shown in 11 patients with peripheral vascular disease (PVD) and 16 normal subjects (NL) [20]. Rest, exercise, and perfusion reserve were measured by contrast-enhanced perfusion MRI of the calf in NL and patients with PVD. The authours concluded that although rest measures are reproducible, they are quite low, do not distinguish NL from PVD, and lead to variability in perfusion reserve measures. Exercise tissue function and perfusion index were the most reproducible measures in patients with PVD for use in clinical trials.

### Functionalization of gadolinium metallofullerenes for detecting atherosclerotic plaque lesions by cardiovascular magnetic resonance

This manuscript describes the development of a contrast agent targeting the CD-36 receptor in atherosclerotic plaque [21]. The authors provide description of the manufacture and evaluation of the agent in Gd-containing endohedrals. They show the time-dependent variation of uptake of the agent in ApoE knockout mice and compare that to wild type mice. A series of detailed and methodical experiments was provided to prepare and characterize the compounds used and to analyze their properties and distribution.

### Rapid prototyping compliant arterial phantoms for in-vitro studies and device testing

This work describes a method of 3D printing to create compliant vascular phantoms for use in CMR experimental studies [22]. The descriptions included two models, the first being a patient-specific compliant hypoplastic aorta suitable for connection in a mock circulatory loop for *in vitro* tests and the second, a right ventricular outflow tract (RVOT) of a patient in need of pulmonary valve replacement. This second model was printed in order to physically test device insertion and assess patient’s suitability for percutaneous pulmonary valve intervention. Measured values of distensibility, compared with literature data, show that the approach is suitable for manufacturing arterial phantoms with representative anatomical finishing, and quick and inexpensive fabrication.

### Evolution of cardiac and renal impairment detected by high-field cardiovascular magnetic resonance in mice with renal artery stenosis

Renal artery stenosis (RAS) is an important underlying cause of hypertension and cardiac dysfunction; the authors used CMR to follow progressive cardiac and renal dysfunction in mice with RAS [23]. Operated and control mice were followed for 4 weeks. In the diseased kidney, renal blood flow declined and BOLD showed development of renal hypoxia. RAS animals also showed evidence of LV hypertrophy with signs of early systolic and diastolic dysfunction. CMR can be used to follow progressive cardiac and renal changes in the murine RAS model.

### Fast retrospectively triggered local pulse-wave velocity measurements in mice with CMR-microscopy using a radial trajectory

Aortic pulse-wave velocity (PWV) is an important determinant of cardiovascular risk. Previous techniques for measurement of PWV in mice required cardiac and respiratory gating. The authors developed a robust self-gated method to measure PWV without the need for triggering [24]. This method will be useful for the study of transgenic mice with altered vascular function.

## Diffusion tensor imaging

Diffusion tensor imaging (DTI) gives information on the mean intravoxel alignments of tissue microstructures using diffusion weighted imaging. It is used in particular in the brain, where larger diffusion values occur along nerve fibres in comparison to across fibres. The technique can also be used with considerably increased difficulty (due to motion) for the heart, [25] however most work has been done in animals, [26] or in ex-vivo human hearts, because of the high difficulty of deriving DTI data from the moving heart. Recently a more robust imaging sequence for in-vivo human cardiac DTI has been described which makes human studies more approachable [27]. Recent work has shown in-vivo reproducibility in humans in hypertrophic cardiomyopathy, [28] and histological validation in animals [29].

### Correction: Reproducibility of *in vivo* diffusion tensor cardiovascular magnetic resonance in hypertrophic cardiomyopathy

The authors had previously reported the reproducibility of using a stimulated-echo single-shot-EPI sequence with zonal excitation and parallel imaging in 10 patients with HCM scanned on 2 different days.^28^ This manuscript represents a correction to an error in that paper [30]. The previous statement that results showed good reproducibility of fractional anisotropy, mean diffusivity and helical angle, indicating that current technology yields robust *in vivo* measurements that have potential clinical value is not changed by this correction. The interpretation of regional differences in the septum requires further investigation.

## Congenital heart disease

CMR of congenital heart disease is relatively mature, although quality acquisition and interpretation require training. CMR is routinely used to investigate congenital heart disease in children and adults, as a complement to echocardiography, to avoid imaging with ionising radiation, reduce the need for invasive diagnostic cardiac catheterisation. Three dimensional data acquisition is usual and approaches for both 3D balanced SSFP and magnetic resonance angiography are improving [31]. Aortopathy associated with congenital or inherited heart diseases is common and the potential for CMR to triage patients is of interest. There has been considerable recent interest in the use of 4D flow imaging, which allows the visualisation of large scale vorticity and the retrospective measurement of flow in vessels within the volume covered [32,33].

### Guidelines and protocols for cardiovascular magnetic resonance in children and adults with congenital heart disease: SCMR expert consensus group on congenital heart disease.

CMR for congenital heart disease has an increasingly valued role for routine diagnostic assessment, serial clinical surveillance and pre procedural planning. The authors of this paper are experienced and expert in the field and give helpful guidelines and consensus recommendations for the performance of CMR in children and adults with congenital heart disease [34]. Common core techniques are discussed and disease specific protocols recommended.

### Demonstration of value of optimizing ECG triggering for cardiovascular magnetic resonance in patients with congenital heart disease

The authors investigated blood flow quantification in 35 patients with varied congenital heart disease using both a new ECG trigger algorithm and an old EGG trigger algorithm [35]. Increased specificity, sensitivity and accuracy of QRS detection was found with the new ECG triggering algorithm. Differences of more than 5% blood flow quantification were seen in approximately 1/3 of cases supporting that optimization of ECG triggering during routine CMR is needed in routine congenital heart disease CMR.

### Preliminary experience with cardiovascular magnetic resonance in evaluation of fetal cardiovascular anomalies

A relatively large contemporary clinical experience is reported of fetal CMR in 68 pregnant women studied at mean gestational age 25.5 weeks for further clarification of congenital heart disease [36]. CMR yielded the same diagnosis as postnatal findings in 79%. Fetal cardiac contractility and valvular function cannot be assessed and small defects were sometimes missed; nevertheless this preliminary experience suggests that fetal CMR may be a promising future assessment tool.

### Fetal circulation in left-sided congenital heart disease measured by cardiovascular magnetic resonance: a case–control study

Interest is growing in the ability of CMR to elucidate pathophysiological mechanisms and in future to evaluate abnormalities of anatomy of function not readily depicted with fetal echocardiography. The relationships between fetal haemodynamics and lung and brain development were examined by Al Nafisi and colleagues. Phase contrast CMR in 22 fetuses with suspected left sided lesions at a single late gestation timepoint (around 35 weeks) was used to determine fetal blood flow distribution and values compared with their previously reported 12 normal controls [37]. Fetuses with left-sided CHD had a mean combined ventricular output 19% lower than normal controls. Fetal brain weight below the 5th centile was seen only in CHD fetuses (n=6) [38]. The authors suggest that the mechanisms of fetal developmental delay in congenital heart disease are a priority for study and that future studies combining fetal brain MRI and phase contrast CMR could contribute.

### PINOT NOIR: Pulmonic INsufficiency imprOvemenT with Nitric Oxide Inhalational Response

Sixteen patients with at least moderate pulmonary regurgitation on echocardiogram with either repaired tetralogy of Fallot (n=11) or previous surgery for pulmonary stenosis (n=5) were prospectively studied with CMR before and during 40ppm inhaled nitric oxide [39]. At baseline, mean pulmonary regurgitant fraction was 35+/−16% and mean RVEDVi and RVESVi were 157+/−33 and 93+/−20 mL/m^2^, respectively. Nitric oxide reduced pulmonary regurgitant fraction by an average of 5+/−8%. However, there was no reduction in right ventricular volume indices which are currently used to help determine timing of pulmonary valve replacement. The longer term effects of selective pulmonary vasodilator therapy might be of interest.

### Inflow-weighted pulmonary perfusion: comparison between dynamic contrast-enhanced MRI versus perfusion scintigraphy in complex pulmonary circulation

Pulmonary scintigraphy is the current convention when assessing regional pulmonary perfusion. In contrast to Technetium labelled macroaggregated albumin, gadolinium chelate is only nanometers in size so can pass freely into capillaries to the systemic circulation and recirculate in normal lungs or abnormally via shunt/collateral flow in pathological lungs. Where present these abnormalities can cause discrepancy between conventional indicator dilution and inflow-weighted analysis methods because the discrepancy represents a mixed-flow component in which pathological flow such as shunting or collaterals might have participated. Based on 26 retrospectively analysed data sets in 22 patients that underwent pulmonary scintigraphy and dynamic contrast enhanced MR as part of another research study, the authors demonstrate a method for CMR-derived inflow weighted regional pulmonary perfusion maps that results in a close match to the gold standard pulmonary scintigraphy [40]. It appears that dynamic contrast enhanced MRI using both conventional indicator dilution theory combined with the newly proposed inflow-weighted analysis could be an attractive substitute for pulmonary scintigraphy in pediatric patients where avoidance of ionising radiation is highly desirable.

## Flow

The measurement of flow using CMR has become legion in the cardiovascular system reflecting its central importance. While some technical issues still need to be addressed to overcome to achieve consistency between manufacturers and centres, [41] recent applications have included left atrial appendage emptying, [42] aortic wave intensity analysis, [43] and robust cardiac output measurement [44].

### Assessment of ductal blood flow in newborns with obstructive left heart lesions by cardiovascular magnetic resonance

Direct CMR measurement of direction and volume of ductal flow was feasible and analysed in 32 newborn patients with left sided obstructive heart disease [45]. Patients with smaller left ventricles and lower ascending aortic flow had a greater contribution of ductal flow to the systemic circulation. The size of the ductus arteriosus did not predict net ductal flow. Comparison with contemporaneous echocardiography was not made in this retrospective study.

## Aorta

CMR is widely used for assessment of the aorta in both congenital and acquired conditions and is particularly well suited longitudinal follow-up of aortic dimensions, and more complex aspects of aortic function, such as pulse wave velocity, distensibility and shear stress [46-48].

### Cardiovascular Magnetic Resonance in Marfan syndrome

This is a comprehensive overview of the cardiovascular manifestations of Marfan syndrome and the role of CMR in their evaluation [49]. The paper is nicely illustrated and addresses an important but often under-appreciated disorder.

### Aortic arch shape is not associated with hypertensive response to exercise in patients with repaired congenital heart diseases

Exercise induced hypertension is a complication of successful coarctation repair and has been previously suggested to be due to a gothic arch morphology post repair. Ntsinjana and colleagues studied 60 subjects (20 controls, 20 post repair of coarctation and 20 post arterial switch operation for transposition of the great arteries) with bicycle cardiopulmonary exercise testing for blood pressure response to exercise and same day CMR MRA for quantitative geometrical analysis [50]. Arch curvature was similar in post coarctation and arterial switch patients but different from controls. Transverse arch and isthmus hypoplasia, not acute arch angulation (“gothic arch”) were associated with pathophysiological blood pressure response to exercise. These data suggest attention should be paid to the size of the residual arch at interventions as this is at least one factor contributing to the abnormal blood pressure response.

### Prediction of aortic dilation in Turner syndrome - enhancing the use of serial cardiovascular magnetic resonance

Women with Turners may develop aortic dilatation of the ascending or descending aorta and are at 100 fold increased risk of aortic dissection. Known risk factors fail to predict dissection in an important proportion. Towards future optimization of triage of these patients, in this study adult women with Turners (n=102) were prospectively followed with CMR over 5 years with aortic assessment at 9 positions and 3 time points (baseline, mean 2.4 years and at mean 4.8 years) [51]. Mathematical models integrating all measurements at all timepoints as well as known risk factors for aortic disease were made to forecast aortic diameter. Aortic coarctation (P < 0.0001), bicuspid aortic valve (P < 0.0001), advanced age (P < 0.0001), diastolic blood pressure (P = 0.0008), body surface area (P = 0.015) and antihypertensive treatment (P = 0.005) all preferentially accelerated aortic diameter. With the model it was possible to identify Turners patients at low and high risk for rapidly progressive aortic growth.

## T1 mapping & extracellular volume

Myocardial assessment by T1 mapping is the technique of the moment, and there has been a large expansion in papers published on this subject in JCMR and elsewhere as its robustness and clinical role becomes defined [52-56].

### Myocardial T1 mapping and extracellular volume quantification: a Society for Cardiovascular Magnetic Resonance (SCMR) and CMR Working Group of the European Society of Cardiology consensus statement

This consensus statement addresses the rapid innovations in CMR enabling the tracking of biologically important changes in the myocardium by: a) native T1 that reflects myocardial disease involving the myocyte and interstitium without use of gadolinium based contrast agents (GBCA), or b) the extracellular volume fraction (ECV)–a direct GBCA-based measurement of the size of the extracellular space, reflecting interstitial disease [57]. The latter technique attempts to dichotomize the myocardium into its cellular and interstitial components with estimates expressed as volume fractions. This document provides recommendations for clinical and research T1 and ECV measurement, based on published evidence when available and expert consensus when not. The authors discuss site preparation, scan type, scan planning and acquisition, quality control, visualisation and analysis, technical development. The authors highlight the need for more research before a large-scale application for clinical decision-making can be recommended.

### Standardization of T1 measurements with MOLLI in differentiation between health and disease – the ConSept study

In this study, the authors highlight that T1 imaging based on pixel-wise quantification of longitudinal relaxation has the potential to differentiate between normal and abnormal myocardium but that the accuracy of T1 measurement has not been established nor systematically tested in the presence of health and disease [58]. Intra-observer, inter-observer and inter-study reproducibility of T1 imaging was assessed in subjects with left ventricular hypertrophy (LVH, n = 25) or dilated cardiomyopathy (DCM, n = 43). Thirty-eight subjects with low-pretest likelihood of cardiomyopathy served as a control group. T1 values were acquired in a single mid-ventricular short axis slice using modified Look-Locker imaging prior and after the application of gadolinium contrast at 1.5 and 3 T. Analysis was performed with regions of interest (ROI) placed conservatively within the septum or to include the whole short axis (SAX) myocardium. Conservative septal ROI T1 measurement was found to be a robust technique with good intra-observer, inter-observer and inter-study reproducibility for native and post-contrast T1 value and partition coefficient measurements. An important conclusion the authors make is that native septal T1 values revealed the greatest difference between normal and abnormal myocardium, which is independent of geometrical alterations of cardiac chamber and wall thickness. Accordingly, they propose the use of native T1 measurements using conservative septal technique as the standardized approach to distinguish health from disease assuming diffuse myocardial involvement.

### Normal variation of magnetic resonance T1 relaxation times in the human population at 1.5 T using ShMOLLI

In this study, the authors aimed to establish the normal range, potential sources of error and confounders of native myocardial T1 relaxation times in a large cohort of healthy human volunteers [59]. T1-mapping was performed at 1.5T using the Shortened Modified Look-Locker Inversion Recovery (ShMOLLI) technique. They reported the effects of normal physiologic parameters, including age, gender, heart rate, weight, height and hematocrit, as well as technical factors, such as partial volume effects, myocardial thickness and inter-centre variability on identical scanners within and across three clinical centres in two countries. A large cohort of healthy volunteers (n = 342), from 3 clinical centres across two countries underwent CMR at 1.5T. Native myocardial ShMOLLI T1 was 962 ± 25 ms. They identified the partial volume as primary source of potential error in the analysis of respective T1 maps and use 1 pixel erosion to represent “midwall myocardial” T1, resulting in a 0.9% decrease to 953 ± 23 ms. Midwall myocardial ShMOLLI T1 was reproducible with an intra-individual, intra- and inter-scanner variability of ≤2%. The principle biological parameter influencing myocardial ShMOLLI T1 was the female gender. After correction for age and gender dependencies, heart rate was the only other physiologic factor with a small effect on myocardial ShMOLLI T1. Left and right ventricular blood ShMOLLI T1 correlated strongly with each other and also with myocardial T1. Overall, the effect of all variables on myocardial ShMOLLI T1 was within 2% of relative changes from the average. They concluded that native T1-mapping using ShMOLLI generates reproducible and consistent results in normal individuals within 2% of relative changes from the average. The main potential confounder is the partial volume effect arising from over-inclusion of neighbouring tissue at the manual stages of image analysis. In the study of cardiac conditions such as diffuse fibrosis or small focal changes, the use of “myocardial midwall” T1, age and gender matching, and compensation for heart rate differences may all help to improve the method sensitivity in detecting subtle changes. ShMOLLI is a stable and reproducible method for T1-mapping.

### Myocardial T1 and T2 mapping at 3 T: reference values, influencing factors and implications

T1 and T2 mapping of the left ventricular myocardium, i.e. quantification of the myocardial T1 and T2 relaxation times, as well as the T1-derived extracellular volume fraction have been demonstrated to add valuable information. Most of the experience with myocardial mapping was gained at a magnetic field strength of 1.5 T. Parametric myocardial mapping at 3 T is conceptually appealing due to the signal gain inherent to higher fields, which may be exploited for improved spatial and temporal resolution. Many of the previous studies focused on intra-individual comparison of diseased and remote myocardium. However, T2 and T1 reference values of all myocardial segments may be important to define small focal abnormalities and to identify diffuse tissue changes in the absence of healthy “remote” myocardium. This study aimed at analyzing the feasibility of T1 and T2 mapping at 3 T and providing reference values [60]. Sixty healthy volunteers (30 males/30females, with 20 each from 20–39 years, 40–59 years, 60–80 years) underwent left-ventricular T1 and T2 mapping in 3 short-axis slices at 3 T. For T2 mapping, 3 single-shot steady-state free precession (SSFP) images with different T2 preparation times were acquired. For T1 mapping, modified Look-Locker inversion recovery technique with 11 single shot SSFP images was used before and after injection of gadolinium contrast. T1 and T2 relaxation times were quantified for each slice and each myocardial segment. Myocardial T2 and T1 reference values for the specific CMR setting are provided. The authors observe that the diagnostic impact of the high inter-subject variability of T2 and T1 relaxation times requires further investigation.

### Diffuse myocardial fibrosis by T1-mapping in children with subclinical anthracycline cardiotoxicity: relationship to exercise capacity, cumulative dose and remodeling

Despite the effectiveness of anthracycline chemotherapy, cardiotoxicity remains a significant long-term secondary effect. The myofibrillar loss and cellular necrosis from anthracycline cardiotoxicity leads to delayed and irreversible myocardial damage, culminating in cardiac dysfunction, cardiomyopathy and heart failure. The aim of this study was to determine the CMR tissue characteristics (myocardial T1, T2 and derived ECV) in subclinical myocardial toxicity and their association with LV function and structure, exercise capacity and chemotherapy dose in childhood and adolescent cancer survivors [61]. Thirty patients (15 ± 3 years), at least 2 years following anthracycline treatment, underwent CMR, echocardiography, and cardiopulmonary exercise testing (peak VO_2_). CMR measured ventricular function, mass, T1 and T2 values, and myocardial extracellular volume fraction, ECV, a measure of diffuse fibrosis based on changes in myocardial T1 values pre- and post-gadolinium. Cardiac function was also assessed with conventional and speckle tracking echocardiography. Patients had normal LVEF (59 ± 7%) but peak VO_2_ was 17% lower than age-predicted normal values and were correlated with anthracycline dose (r = −0.49). Increased ECV correlated with decreased mass/volume ratio (r = −0.64), decreased LV wall thickness/height ratio (r = −0.72), lower peak VO_2_(r = −0.52), and higher cumulative dose (r = 0.40). Echocardiographic measures of systolic and diastolic function were reduced compared to normal values (p < 0.01), but had no relation to ECV, peak VO_2_ or cumulative dose. Myocardial T1 and ECV were found to be early tissue markers of ventricular remodeling that may represent diffuse fibrosis in children with normal ejection fraction post anthracycline therapy, and are related to cumulative dose, exercise capacity and myocardial wall thinning.

### Modified look-locker inversion recovery T1 mapping indices: assessment of accuracy and reproducibility between magnetic resonance scanners

This study presents results on the accuracy and reproducibility of MOLLI T1 measurements on two different MR manufactured systems each at two different field strengths 1.5 Tesla and 3 Tesla [62]. The T1 of Gel phantoms is systematically measured and repeated on multiple MR systems for heart rates from 40 to 100 beats per min. Results are compared to those from the gold standard IR-SE T1 sequence. Errors in T1 are also compared to errors in partition coefficient as calculated from combined measurements in phantoms with a high and a low T1 and the results show that the range of T1 errors is higher than that of errors for the partition coefficient.

### Influence of off-resonance in myocardial T1-mapping using SSFP based MOLLI method

This manuscript studies the errors in T1 mapping due to properties of the SSFP readout in the approach to steady-state, where the signal intensity depends on both the off-resonance frequency and magnetization preparation [63]. The results show that the most commonly used MOLLI variant with a flip angle of 35 degrees can yield relatively large errors in non-contrast myocardial T1 values. These are important in magnitude given the low variability in the T1 values routinely achieved, which offer the ability to detect pathology with differences in T1. The study also shows that the effects are more pronounced at 3T. Studies such as this will be important in the push to clinically translate these methods.

### T1 and extracellular volume mapping in the heart: estimation of error maps and the influence of noise on precision

The manuscript describes a detailed and accurate method for calculating the error in the T1 measurement on a pixel by pixel basis [64]. The approach used is a standard error analysis method, but it has been applied to these cardiac T1 and ECV mapping data. The method produces Standard Deviation (SD) maps for each T1 and ECV map and the pixel SD estimates are validated by numerical simulation using Monte-Carlo analysis and with phantoms using repeated trials. SD estimates are provided for pre-and post-contrast optimized protocols for a range of T1s and SNRs. The authors conclude that the ability to quantify the measurement error has potential to determine the statistical significance of subtle abnormalities that arise due to diffuse disease processes involving fibrosis and/or edema and is useful both as a confidence metric for overall quality, and in optimization and comparison of imaging protocols.

### Correction with blood T1 is essential when measuring post-contrast myocardial T1 value in patients with acute myocardial infarction

Post-contrast T1 mapping using modified Look-Locker inversion recovery sequence has been introduced as a promising means to assess expansion of the myocardial extra-cellular space even in the absence of scarring. As the degree of left ventricular (LV) myocardial fibrosis can differ from segment to segment, especially in patients with ischemic heart disease, multiple slicing is needed to see regional differences in T1 values. However, post-contrast T1 value can change over time after bolus contrast injection. In this study, the authors investigated the changes of the T1 values according to multiple slicing over scanning time at 15 minutes after contrast injection and usefulness of blood T1 correction [65]. They also evaluated correction methods to provide better time-independent results during multiple short-axis slicing. Eighteen reperfused acute myocardial infarction (AMI) patients, 13 cardiomyopathy patients and 8 healthy volunteers underwent cardiovascular magnetic resonance with 15 minute-post contrast MOLLI to generate T1 maps. In 10 cardiomyopathy cases, pre- and post-contrast MOLLI techniques were performed to generate extracellular volume fraction (Ve). Six slices of T1 maps according to the LV short axis, from apex to base, were consecutively obtained. Each T1 value was measured in the whole myocardium, infarcted myocardium, non-infarcted myocardium and LV blood cavity. They found that post-contrast 15-minute myocardial T1 value increased over time but the ratio of myocardial T1/blood T1 was rather stable. The Ve calculated using pre-, post- MOLLI sequence and blood hematocrit level, was stable throughout the entire myocardial region. They recommend using 15-minute-post-contrast myocardial T1 values after correcting for blood T1 or calculated Ve when multiple slicing is conducted over time.

## Interventional CMR

Few subjects create as much interest clinically as the potential for replacement of x-ray based interventional procedures by those under MR guidance. The design of interventional devices has continued to advance, [66-68] and the field was recently reviewed [69].

### Real-time cardiovascular magnetic resonance subxiphoid pericardial access and pericardiocentesis using off-the-shelf devices in swine

This study assesses whether interventional CMR could allow enhanced safer image guidance for needle access for drainage of pericardial effusion [70]. Traditional guidance procedures using 2D projection angiography or ultrasound, carry the risk that the needle location and trajectory can be misjudged potentially resulting in serious complications or even death. This study uses real-time CMR to guide subxiphoid pericardial access in naïve swine using commercial 18G titanium puncture needles, which are exchanged for pericardial catheters. To test the value of CMR needle pericardiocentesis, the authors also created intentional pericardial effusions of a range of volumes, via a separate transvenous-transatrial catheter. The authors concluded that the experience from this study supports clinical testing of real-time CMR guided needle access or drainage of the pericardial space.

### Transthoracic delivery of large devices into the left ventricle through the right ventricle and interventricular septum: preclinical feasibility

In 9 of 11 pigs, real time CMR guidance successfully allowed LV cannulation via the chest, RV free wall and the interventricular septum, and subsequent closure of the RV free wall with an occluder device; one failure was due to refractory ventricular fibrillation and one from inadequate guidewire support [71]. The septum recoiled immediately and complete healing was demonstrated with oximetry, angiography, CMR and necropsy up to 4 weeks later. The entry angle for accessing the left sided chambers is more favourable than the conventional atrial transseptal approach which may be attractive when considering transcatheter mitral valve interventions.

### Pediatric cardiovascular interventional devices: effect on CMR images at 1.5 and 3 Tesla

Using ex-vivo phantom experiments, 11 different pediatric transcatheter devices (stainless steel or nitinol) were evaluated at 1.5 and 3.0 Tesla for the influence of magnetic field strength, pulse sequence type, device orientation, flip angle and voxel size on the type and extent of CMR artifact [72]. Stainless steel embolization coils rendered large surrounding zones uninterpretable whilst remaining devices cause mild artifact. The strength of the magnetic field, device composition, sequence type and orientation of the device in B0 had the largest impact on the amplification factor (ratio of signal void dimension to true device dimension) of implanted devices in an MR environment

## Cardiomyopathy

The phenotyping of cardiomyopathy is a primary clinical indication for CMR, [73] and has become mainstream in hypertrophic cardiomyopathy [74-77]. Recent attention has been focussed on the assessment of diffuse fibrosis in cardiomyopathy using T1 imaging, [78,79] and novel investigations in less common conditions [80-83].

### Left ventricular noncompaction in Duchenne muscular dystrophy

In this study, the authors report on left ventricular noncompaction (LVNC) in the Duchenne Muscular Dystrophy (DMD) population and characterize its relationship to global LV function [84]. CMR was used to assess ventricular morphology and function in 151 subjects: DMD with ejection fraction (EF) > 55% (n = 66), DMD with EF < 55% (n = 30), primary LVNC (n = 15) and normal controls (n = 40). The non-compacted to compacted (NC/C) ratio was measured in each of the 16 standard myocardial segments. LVNC was defined as a diastolic NC/C ratio > 2.3 for any segment. LVNC criteria were met by 27/96 DMD patients (prevalence of 28%): 11 had an EF > 55% (prevalence of 16.7%), and 16 had an EF < 55% (prevalence of 53.3%). The median maximum NC/C ratio was 1.8 for DMD with EF > 55%, 2.46 for DMD with EF < 55%, 1.54 for the normal subjects, and 3.69 for primary LVNC patients. Longitudinal data for 78 of the DMD boys demonstrated a mean rate of change in NC/C ratio per year of +0.36. The high prevalence of LVNC in DMD was associated with decreased LV systolic function that develops over time and may represent muscular degeneration versus compensatory remodeling.

### Prevalence and distribution of late gadolinium enhancement in a large population of patients with Duchenne muscular dystrophy: effect of age and left ventricular systolic function

Duchenne muscular dystrophy (DMD), an X-linked recessive disorder affecting approximately 1 in 5000 males, is the most common inherited muscular dystrophy. The disease results from mutations in the gene for dystrophin, a sarcolemmal protein that is abundant in both cardiac and skeletal muscle. Progressive skeletal muscle weakness results in loss of ambulation between 7 and 13 years of age. DMD-associated cardiac disease is progressive and ultimately results in global ventricular systolic dysfunction, often with minimal ventricular dilation. End-stage cardiac pathology includes cardiomyocyte hypertrophy, atrophy and fibrosis. In this study, the authors sought to establish a) prevalence and distribution of LGE in a large DMD population and b) relationship among LGE, age, LVEF by CMR and current living status [85]. They demonstrate that LGE occurs early, is progressive and increases with both age and decreasing LVEF. Segmentally, the incidence of the number of positive LGE segments increase with age and lower LVEF. Older patients and those who died during the study period had more septal LGE involvement. The current studies suggest that the time course and distribution of LGE-positivity may be an important clinical biomarker to aid in the management of DMD-associated cardiac disease.

### Arrhythmogenic right ventricular cardiomyopathy mimics: role of cardiovascular magnetic resonance

CMR is commonly used in patients with suspected arrhythmogenic right ventricular cardiomyopathy (ARVC). However, various diseases may present with clinical features resembling ARVC causing diagnostic dilemmas. This study explored the role of CMR in the differential diagnosis of 657 patients with suspected ARVC. Twenty patients (3.0%) fulfilled imaging ARVC criteria [86]. Thirty (4.6%) had a potential ARVC mimic, of which 25 (3.8%) were considered clinically important. In conclusion, some patients referred for CMR with suspected ARVC fulfil ARVC imaging criteria but more have otherwise unrecognised diseases mimicking potentially ARVC. Clinical assessment should emphasize the assessment of potential mimics in parallel with the detection of ARVC.

### Pulmonary blood volume indexed to lung volume is reduced in newly diagnosed systemic sclerosis compared to normals – a prospective clinical cardiovascular magnetic resonance study addressing pulmonary vascular changes

Pulmonary involvement, either by pulmonary arterial hypertension or pulmonary fibrosis, is the most common cause of death in systemic sclerosis (SSc). The authors explored the feasibility of detecting early pulmonary involvement in SSc using non-invasive quantitative measures of pulmonary physiology by CMR. Compared to healthy controls, the pulmonary blood volume (PBV) indexed to lung volume (PBVI) was lower in newly diagnosed SSc patients. There was no significant correlation between PBVI and pulmonary artery pressure estimated by Doppler, lung’s diffusion capacity for carbon monoxide, vital capacity, or pulmonary fibrosis by CT. Hence PBVI may be a novel parameter reflecting vascular lung involvement in early-stage SSc [87].

### Varied distributions of late gadolinium enhancement found among patients meeting cardiovascular magnetic resonance criteria for isolated left ventricular non-compaction

The aim of this retrospective study was to describe the frequency and distribution of LGE in patients considered to meet CMR cine imaging criteria for LVNC [88]. Forty-seven patients meeting standard CMR criteria for LVNC were studied. Mean number of non-compacted segments per patient was 7.4 ± 2.5 and the NC:C was 3.2 ± 0.7. Non-compaction was most commonly noted in the apical segments in all patients. LGE was present in 40%, and most often located in the ventricular septum. The distribution of LGE was subendocardial (n = 5; 6%), mid-myocardial (n = 61; 68%), subepicardial (n = 10; 11%), and transmural (n = 14; 15%) in total of 90 LGE (+) segments. LGE distribution was noted to be heterogeneous with appearances potentially attributable to three or more distinct cardiomyopathic processes. The authors observe that further work is needed to determine whether conditions such as dilated cardiomyopathy, previous myocarditis or ischaemic heart disease increase the apparent depth of non-compact relative to compact myocardium.

### Quantification of left ventricular trabeculae using fractal analysis

The identification by imaging of left ventricular non-compaction (LVNC) is challenging and relatively subjective. Captur and colleagues report a novel approach: the measurement of the fractal dimension (FD) of the LV endocardial border [89]. This dimension, indicative of boundary complexity or fragmentation, was found to be higher in 30 patients assessed by the Jenni echocardiographic criteria as having LVNC compared to healthy volunteers. It was also higher in healthy blacks (n=30) than healthy whites (n=36). Fractal analysis, which is likely to be dependent on image resolution, was considered to provide a quantitative measure of trabeculation with high reproducibility and accuracy for LVNC diagnosis as compared to previous CMR criteria based on the linearly measured proportions of trabeculated to compact layers as seen in long axis cines.

### Heterogeneous abnormalities of in-vivo left ventricular calcium influx and function in mouse models of muscular dystrophy cardiomyopathy

Manganese-enhanced CMR (MECMR) can non-invasively assess myocardial calcium influx, and in this study, the authors examined whether myocardial calcium levels are elevated in vivo in two mouse models of muscular dystrophy cardiomyopathy, the mdx mice (model of Duchenne Muscular Dystrophy) and Sgcd−/− mice (Limb Girdle Muscular Dystrophy) [90]. Both mouse models exhibited increased in-vivo calcium influx at an early stage in the development of the cardiomyopathy before left ventricular hypertrophy occurred. This study shows the capability of MECMR to measure calcium fluxes in vivo.

### On myocardial siderosis and left ventricular dysfunction in hemochromatosis

Chronically increased intestinal iron uptake in genetic hemochromatosis (HC) may cause organ failure. Whilst iron loading from blood transfusions may cause dilated cardiomyopathy in conditions such as thalassemia, the in-vivo prevalence of myocardial siderosis in HC is unclear, and its relation to left ventricular (LV) dysfunction is controversial. Most previous data on myocardial siderosis in HC has come from post-mortem studies. In this paper, T2* CMR was performed at first presentation of 41 HC patients (58.9 ± 14.1 years) to measure myocardial iron and left ventricular (LV) ejection fraction (EF). In 31 patients (genetically confirmed HFE-HC), the HFE genotype was C282Y/C282Y (n = 30) and C282Y/H63D (n = 1) [91]. Patients with other genotypes (n = 10) were labeled genetically unconfirmed HC. Of the genetically confirmed HFE-HC patients, 6 (19%) had myocardial siderosis (T2* <20 ms). Of these, 5 (83%) had heart failure and reduced LVEF which was correlated to the severity of siderosis (R2 0.57, p = 0.049). Two patients had follow-up scans and both had marked improvements in T2* and LVEF following venesection. Myocardial siderosis was present in 6/18 (33%) of patients with presenting ferritin ≥ 1000 μg/L at diagnosis but in 0/13 (0%) patients with ferritin <1000 μg/L (p = 0.028). Overall however, the relation between myocardial siderosis and ferritin was weak (R2 0.20, p = 0.011). In the 10 genetically unconfirmed HC patients, 1 patient had mild myocardial siderosis but normal EF. Of all 31 patients, 4 had low LVEF from other identifiable causes without myocardial siderosis. The authors concluded: myocardial siderosis was present in 33% of newly presenting genetically confirmed HFE-HC patients with ferritin >1000 μg/L, and was the commonest cause of reduced LVEF; Heart failure due to myocardial siderosis was only found in these HFE-HC patients, and was reversible with venesection; and that myocardial iron was normal in patients with other causes of LV dysfunction.

### Cardiac and hepatic iron and ejection fraction in thalassemia major: multicentre prospective comparison of combined deferiprone and deferoxamine therapy against deferiprone or deferoxamine monotherapy

Due to the limited data available in literature, the aim of this multi-centre study was to prospectively compare in thalassemia major (TM) patients the efficacy of combined deferiprone (DFP) and deferoxamine (DFO) regimen versus either DFP and DFO in monotherapy by CMR (CMR) over a follow up of 18 months. Among the first 1135 TM patients in the MIOT (Myocardial Iron Overload in Thalassemia) network, those who had received either combined regimen (DFO + DFP, N=51) or DFP (N=39) and DFO (N=74) monotherapies were evaluated between the two CMR scans. Iron overload was measured by T2* multiecho technique [92]. Biventricular function parameters were quantitatively evaluated by cine images. The percentage of patients that maintained a normal global heart T2* value was comparable between DFP+DFO versus both monotherapy groups. Among the patients with myocardial iron overload at baseline, the changes in the global heart T2* and in biventricular function were not significantly different in DFP+DFO compared with the DFP group. The improvement in the global heart T2* was significantly higher in the DFP+DFO than the DFO group, without a difference in biventricular function. Among the patients with hepatic iron at baseline, the decrease in liver iron concentration values was significantly higher with combination therapy than with either monotherapy group. The authors concluded that in TM patients at the dosages used in the real world, the combined DFP+DFO regimen was more effective in removing cardiac iron than DFO, and was superior in clearing hepatic iron than either DFO or DFP monotherapy. Combined therapy did not show an additional effect on heart function over DFP.

### Right and left ventricular function and myocardial scarring in adult patients with sickle cell disease: a comprehensive magnetic resonance assessment of hepatic and myocardial iron overload

Patients with Sickle cell disease (SCD) who receive regular transfusions are at risk for developing cardiac toxicity from iron overload. The aim of this study was to assess right and left cardiac volumes and function, late gadolinium enhancement (LGE) and iron deposits in patients with SCD using CMR, correlating these values with transfusion burden, ferritin and hemoglobin levels [93]. Thirty patients with SCD older than 20 years of age were studied in a 1.5 T scanner and compared to age- and sex-matched normal controls. Patients underwent analysis of biventricular volumes and function, LGE and T2* assessment of the liver and heart. When compared to controls, patients with SCD presented higher left ventricular (LV) volumes with decreased ejection fraction (EF) with an increase in stroke volume (SV) and LV hypertrophy. The right ventricle (RV) also presented with a decreased EF and hypertrophy, with an increased end-systolic volume. Although twenty-six patients had increased liver iron concentrations (median liver iron concentration value was 11.83 ± 9.66 mg/g), only one patient demonstrated an abnormal heart T2* < 20 msec. Only four patients (13%) had LGE, with only one patient with an ischemic pattern. The authors concluded that abnormal heart iron levels and myocardial scars are not a common finding in SCD despite increased liver iron overload. The significantly different ventricular function seen in SCD compared to normal suggests the changes in RV and LV function may not be due to the anemia alone. Future studies are necessary to confirm this association.

### Understanding cardiovascular injury after treatment for cancer: an overview of current uses and future directions of cardiovascular magnetic resonance.

Cancer-free survival has improved over the past 20 years for many individuals with prostate, renal, breast, and hematologic malignancies, but an increasingly recognized prevalence of cardiovascular (CV) events in cancer survivors has been an unintended consequence of many of the therapies that have improved these survival rates. The increase in CV events threatens to offset the improvement in cancer related survival. As a result, there is an emerging need to develop methods to identify those individuals treated for cancer at increased risk of cardiovascular events. With its inherent ability to characterize myocardial tissue and identify both cardiac and vascular dysfunction, CMR has the potential to identify both subclinical and early clinical CV injury before the development of an overt catastrophic event such as a myocardial infarction, stroke, or premature cardiac death. Early identification provides an opportunity for the implementation of primary prevention strategies to prevent such events, thereby improving overall cancer survivorship and quality of life. This article reviews the etiology of CV events associated with cancer therapy and the unique potential of CMR to provide early diagnosis of subclinical CV injury related to the administration of these therapies [94].

### Treatment of heart failure in adults with thalassemia major: response in patients randomised to deferoxamine with or without deferiprone.

Established heart failure in thalassaemia major has a poor prognosis and optimal management remains unclear. This was a 1 year prospective study comparing deferoxamine (DFO) monotherapy or when combined with deferiprone (DFP) for patients with left ventricular ejection fraction (LVEF) <56% [95]. All patients received DFO at 50–60 mg/kg 12–24 hr/day sc or iv 7 times weekly, combined with either DFP 75 at mg/kg/day (combination arm) or placebo (DFO monotherapy arm). The primary endpoint was the change in LVEF by CMR. Improvement in LVEF was significant in both study arms at 6 and 12 months (p = 0.04), normalizing ventricular function in 9/16 evaluable patients. With combination therapy, the LVEF increased from 49.9% to 55.2% (+5.3% p = 0.04; n = 10) at 6 months and to 58.3% at 12 months (+8.4% p = 0.04; n = 7). With DFO monotherapy, the LVEF increased from 52.8% to 55.7% (+2.9% p = 0.04; n = 6) at 6 months and to 56.9% at 12 months (+4.1% p = 0.04; n = 4). The LVEF trend did not reach statistical difference between study arms (p = 0.89). In 2 patients on DFO monotherapy during the study and in 1 patient on combined therapy during follow up, heart failure deteriorated fatally. The study was originally powered for 86 participants to determine a 5% difference in LVEF improvement between treatments. The study was prematurely terminated due to slow recruitment and with the achieved sample size of 20 patients there was 80% power to detect an 8.6% difference in EF, which was not demonstrated. Myocardial T2* improved in both arms (combination +1.9 ± 1.6 ms p = 0.04; and DFO monotherapy +1.9 ± 1.4 ms p = 0.04), but with no significant difference between treatments (p = 0.65). Liver iron (p = 0.03) and ferritin (p < 0.001) both decreased significantly in only the combination group. The authors concluded that both treatments significantly improved LVEF and myocardial T2*. Although this is the largest and only randomized study in patients with LV decompensation, further prospective evaluation is needed to identify optimal chelation management in these high-risk patients.

## Valves

The application of CMR to the assessment of valvular heart disease continues to increase, [96] particularly in aortic stenosis [97,98]. This is in part due to greater appreciation of its complementary roles in relation to echocardiography, which is commonly the first line investigative technique.

### A novel technique to quantify the instantaneous mitral regurgitant rate

Rates of mitral regurgitation are known to be variable through the course of systole. This study set out to quantify such variation by CMR in 41 patients with mitral regurgitation [99]. Systole was divided equally into early, mid, and late systolic parts. Aortic flow and left ventricular stroke volume (LVSV) were plotted against time, so enabling the regurgitant rate to be calculated for each third of systole. Variations of mitral regurgitant rate were found, even among patients with holosystolic mitral regurgitation jets, highlighting the need to take temporal variation into consideration.

### Discrepancies between cardiovascular magnetic resonance and Doppler echocardiography in the measurement of transvalvular gradient in aortic stenosis: the effect of flow vorticity

This study investigated factors, in aortic stenosis (AS), that might underlie discrepancies between CMR and transthoracic echocardiographic (TTE) calculations of transvalvular mean pressure gradient [100]. The effect of vorticity, as calculated from CMR velocity acquisitions, was evaluated in 8 volunteers and 60 patients with AS who had been studied by CMR and TTE. Strouhal number and energy loss were also calculated from CMR velocity data. Although some assumptions underlie the calculations made, the authors concluded that flow vorticity was a major factor underlying the discrepancies of MPG measurement between CMR and TTE.

### Early detection of subclinical ventricular deterioration in aortic stenosis with cardiovascular magnetic resonance and echocardiography

Aortic stenosis (AS) patients with late gadolinium enhancement (LGE) are known to have worse outcome. Using echocardiography and CMR, the authors investigated whether LGE would be useful to detect left ventricular (LV) structural and functional abnormalities in AS. Although there was no difference in aortic valve area between groups, there was a significant trend towards increasing LV volumes, LV mass, worsening of LV diastolic function with the presence of LGE and reduced LV ejection fraction, which coincided with worsening functional capacity. In addition, the amount of myocardial fibrosis on CMR correlated with parameters of diastolic elastance and end-systolic elastance. These findings suggest a potential role of CMR for early detection of subclinical LV structural and functional deterioration in AS patients [101].

### Left ventricular reverse remodeling after transcatheter aortic valve implantation: a cardiovascular magnetic resonance study

CMR was used to investigate the degree of left ventricular mass regression and changes in left ventricular function six months after transcatheter aortic valve implantation (TAVI). As previously reported with surgical aortic valve replacement, left ventricular mass decreased significantly after six months of follow-up. There was a trend for improvement in left ventricular ejection fraction. Left ventricular end-diastolic volume and stroke volume did not change significantly. Based on these findings, significant left ventricular reverse remodeling is expected to occur six months after TAVI [102].

## Myocardial perfusion

Although myocardial perfusion CMR has been possible for many years, the clinical sequence and data processing remain variable between centres. Large multicentre trials are starting to address this issue, [103,104] and technical advances are constantly being made, [105,106] with improved understanding of imaging artefacts [107]. The clinical consideration of best application are also under scrutiny [108,109]. The papers below help to close the gap between research and clinical practice.

### Prognostic value of normal regadenoson stress perfusion cardiovascular magnetic resonance

Regadenoson is a vasodilator stress agent that selectively activates the A2A receptor. Compared to adenosine, regadenoson is easier to administer and results in fewer side effects. Although extensively studied in patients undergoing nuclear perfusion imaging (MPI), its use for perfusion CMR is not well described. The aim of this study was to determine the prognostic value of a normal regadenoson perfusion CMR in patients with known or suspected coronary artery disease [110]. Patients with known or suspected coronary artery disease were prospectively enrolled to receive perfusion CMR (Philips 1.5 T) with regadenoson. Three short-axis slices of the left ventricle (LV) were obtained during first pass of contrast using a hybrid GRE-EPI pulse sequence (0.075 mmol/kg Gadolinium-DTPA-BMA at 4 ml/sec). Imaging was performed 1 minute after injection of regadenoson (0.4 mg) and repeated 15 minutes after reversal of hyperemia with aminophylline (125 mg). Perfusion defects were documented if they persisted for ≥ 2 frames after peak enhancement of the LV cavity. CMR was considered abnormal if there was a resting wall motion abnormality, decreased LVEF (<40%), presence of LGE, or the presence of a perfusion defect during hyperemia. All patients were followed for a minimum of 1 year for major adverse cardiovascular event (MACE) defined as coronary revascularization, non-fatal myocardial infarction, and cardiovascular death. 149 patients were included in the final analysis. Perfusion defects were noted in 43/149 (29%) patients; 59/149 (40%) had any abnormality on CMR. During the mean follow-up period of 24 ± 9 months, 17/149 (11.4%) patients experienced MACE. The separation in the survival distributions for those with perfusion defects and those without perfusion defects was highly significant (log-rank p = 0.0001). When the absence of perfusion defects was added to the absence of other resting CMR abnormalities, the negative predictive value improved from 96% to 99%. The authors concluded that Regadenoson perfusion CMR provides high confidence for excellent prognosis in patients with normal perfusion

### Regadenoson and adenosine are equivalent vasodilators and are superior than dipyridamole- a study of first pass quantitative perfusion cardiovascular magnetic resonance

Regadenoson, dipyridamole and adenosine are commonly used vasodilators in myocardial perfusion imaging for the detection of obstructive coronary artery disease. There are few comparative studies of the vasodilator properties of regadenoson, adenosine and dipyridamole in humans. The specific aim of this study was to determine the relative potency of these three vasodilators by quantifying stress and rest myocardial perfusion in humans using CMR [111]. Fifteen healthy normal volunteers, with Framingham score less than 1% underwent vasodilator stress testing with regadenoson (400 μg bolus), dipyridamole (0.56 mg/kg) and adenosine (140 μg /kg/min) on separate days. Rest perfusion imaging was performed initially. Twenty minutes later, stress imaging was performed at peak vasodilation, i.e. 70 seconds after regadenoson, 4 minutes after dipyridamole infusion and between 3–4 minutes of the adenosine infusion. Myocardial blood flow (MBF) in ml/min/g and myocardial perfusion reserve (MPR) were quantified using a fully quantitative model constrained deconvolution. Regadenoson produced higher stress MBF than dipyridamole and adenosine (3.58 vs 2.81 vs 2.78 ml/min/g, p = 0.0009 and p = 0.0008 respectively). Regadenoson had a much higher heart rate response than adenosine and dipyridamole respectively (9 5vs 76 vs 86 beats/ minute) When stress MBF was adjusted for heart rate, there were no differences between regadenoson and adenosine (37.8 vs 36.6 μl/s/g, p = NS), but differences between regadenoson and dipyridamole persisted (37.8 vs 32.6 μl/s/g, p = 0.03). The unadjusted MPR was higher with regadenoson (3.11) when compared with adenosine (2.7, p = 0.02) and when compared with dipyridamole (2.61, p = 0.04). Similar to stress MBF, these differences in MPR between regadenoson and adenosine were abolished when adjusted for heart rate (2.04 vs 2.12, p = NS), but persisted between regadenoson and dipyridamole (2.04 vs 1.77 , p = 0.07) and between adenosine and dipyridamole (2.12 vs 1.77, p =0.01). The authors concluded that regadenoson and adenosine have similar vasodilator efficacy and are superior to dipyridamole.

### Cost-effectiveness of cardiovascular magnetic resonance and single-photon emission computed tomography for diagnosis of coronary artery disease in Germany

Recent studies have demonstrated a superior diagnostic accuracy of CMR for the detection of coronary artery disease (CAD). The authors aimed to determine the comparative cost-effectiveness of CMR versus single-photon emission computed tomography (SPECT) [112]. Based on Bayes' theorem, a mathematical model was developed to compare the cost-effectiveness and utility of CMR with SPECT in patients with suspected CAD. Invasive coronary angiography served as the standard of reference. Effectiveness was defined as the accurate detection of CAD, and utility as the number of quality-adjusted life-years (QALYs) gained. Model input parameters were derived from the literature, and the cost analysis was conducted from a German health care payer's perspective. Reimbursement fees represented only a minor fraction of the total costs incurred by a diagnostic strategy. Increases in the prevalence of CAD were generally associated with improved cost-effectiveness and decreased costs per utility unit (ΔQALY). By comparison, CMR was consistently more cost-effective than SPECT, and showed lower costs per QALY gained. Given a CAD prevalence of 0.50, CMR was associated with total costs of €6,120 for one patient correctly diagnosed as having CAD and with €2,246 per ΔQALY gained versus €7,065 and €2,931 for SPECT, respectively. Above a threshold value of CAD prevalence of 0.60, proceeding directly to invasive angiography was the most cost-effective approach. The authors concluded that in patients with low to intermediate CAD probabilities, CMR is more cost-effective than SPECT. Moreover, lower costs per utility unit indicate a superior clinical utility of CMR.

### Rapid ungated myocardial perfusion cardiovascular magnetic resonance: preliminary diagnostic accuracy

The authors describe a rapid ungated radial turboFLASH saturation recovery myocardial perfusion sequence at 3T [113]. A hybrid pulse train is used for the saturation followed by 4 to 5 slices of undersampled radial k-space image data rapidly acquired. This acquisition is continuously repeated approximately four times per second and image reconstruction used an iterative constrained method. The ungated perfusion images were post-processed into ungated, self-gated near systolic and self-gated near diastolic images. The method was tested by comparison to quantitative coronary angiography in a small cohort of patients with a mix of sinus rhythm and atrial fibrillation. This simplified ungated radial dynamic CMR perfusion imaging approach was shown to give high quality imaging with high diagnostic accuracy.

### Combined measurement of perfusion, venous oxygen saturation, and skeletal muscle T2* during reactive hyperemia in the leg

This manuscript describes how a pulsed arterial spin labelling method for measuring tissue perfusion can be combined in an interleaved fashion with a multi gradient echo sequence enabling the simultaneous measurement of venous oxygen saturation and T2* for BOLD imaging [114]. The individual methods have been previously developed and validated and in this work the combined sequence is compared and validated against the independent measurements. The method is applied to the assessment of vascular function by imaging during reactive hyperaemia both in healthy volunteers and patients with peripheral artery disease. The novel sequence (given the new acronym PIVOT) achieves excellent temporal resolution, and preserves good spatial resolution via keyhole imaging for the oxygenation components.

### Hyperemic stress myocardial perfusion cardiovascular magnetic resonance in mice at 3 Tesla: initial experience and validation against microspheres

Dynamic first-pass contrast-enhanced myocardial perfusion is the standard CMR method for the estimation of myocardial blood flow in man, but it is challenging in rodents because of the high temporal and spatial resolution requirements. Working at 3T, this is the first report on first-pass stress perfusion imaging in mice [115]. The authors determined absolute blood flow at rest and during dipyridamole stress, as well as perfusion reserve (2.4 ± 0.5), which agreed with literature values. There was also close agreement with microsphere validation. This method has implications for the study of stress-induced ischemia in murine models.

### Oxygenation-sensitive cardiovascular magnetic resonance

Oxygenation-sensitive CMR is a non-contrast technique that allows the non-invasive assessment of myocardial oxygenation. It capitalizes on the fact that deoxygenated hemoglobin in blood can act as an intrinsic contrast agent, changing proton signals in a fashion that can be imaged to reflect the level of blood oxygenation. Increases in oxygen saturation increase the BOLD imaging signal (T2 or T2*), whereas decreases diminish it. This review presents the basic concepts and limitations of the BOLD technique, and summarizes the preclinical and clinical studies in the assessment of myocardial oxygenation with a focus on recent advances [116]. Finally, it provides future directions and a brief look at emerging techniques of this evolving CMR field.

## Acute and chronic coronary artery disease

CMR is invaluable to the study of patients with ischaemic heart disease and has made a major impact on this field and is now widely used to delineate myocardial infarction, [117] although more recently non-contrast techniques to detect infarction have been described [118,119]. The use of native contrast techniques to define myocardial edema, [120,121] myocardium at risk, [122] myocardial hemorrhage, [123,124] and microvascular obstruction, [125] has also grown greatly. Work has moved on to evaluating the relation between myocardial damage and aspects of myocardial function such as dyssynchrony, [126,127] remodelling, [128,129] healing, [130] and novel treatments [131,132]. The papers below show how CMR can be used in a number of ways to characterize acute and chronic coronary disease.

### Incremental value of cardiovascular magnetic resonance over echocardiography in the detection of acute and chronic myocardial infarction

The diagnostic accuracy of echocardiography in detecting ST elevation myocardial infarction was compared to CMR in both acute and chronic stages. Sensitivity of echocardiography to detect acute and chronic myocardial infarctions based on the presence of regional wall motion abnormalities was 78.7% and 61.3%, respectively; specificity was 80.6% in both settings. Echocardiography was therefore more sensitive to detect acute versus chronic infarctions. Undetected myocardial infarctions were smaller, less extensive and less transmural, associated with non-anterior localization and higher ejection fraction as compared to detected myocardial infarctions [133].

### Scar extent evaluated by late gadolinium enhancement CMR: a powerful predictor of long term appropriate ICD therapy in patients with coronary artery disease

The purpose of this study was to compare different methods of quantifying myocardial scar extent and their value as predictors of appropriate ICD therapy in patients with coronary artery disease. Total myocardial scar and percentage of myocardial scar as determined by late gadolinium enhancement were found to be predictors of appropriate discharge. Four different measures of scar transmurality were assessed: 1) “scar transmurality area based”; 2) “scar transmurality line based”; 3) “weighted infarct transmurality”, and; 4) “spatial maximal scar transmurality”. There was some variation between the different measures of transmurality and outcomes, but the number of events was relatively small [134]. Further studies are needed to obtain a consensual method of determining scar transmurality.

### Comparison of diffusion-weighted with T2-weighted imaging for detection of edema in acute myocardial infarction

The aim of this study was to assess myocardial edema with diffusion weighted imaging (DWI) in patients with acute myocardial infarction (AMI) and to compare it with a T2 turbo inversion recovery, black blood sequences - TIRM [135]. The study included 91 acute and post STEMI patients. They applied a qualitative and quantitative image analysis. The qualitative analysis consisted of evaluation of the quality of blood suppression, presence of artifacts and occurrence of high signal (edema) areas. On the basis of edema detection in AMI and control (post STEMI) group, the sensitivity and specificity of TIRM and DWI were determined. Two contrast to noise ratios (CNR) were calculated: CNR1 - the contrast between edema and healthy myocardium and CNR2 - the contrast between edema and intraventricular blood pool. The area of edema was measured for both TIRM and DWI sequences and compared with the infarct size in LGE images. The study indicated that diffusion weighted imaging detects areas of increased myocardial signal in patients with acute myocardial infarction with high sensitivity and specificity (83.1% and 90.0% respectively). DWI may also be used to differentiate chronic from acute injury.

### Scar heterogeneity on cardiovascular magnetic resonance as a predictor of appropriate implantable cardioverter defibrillator therapy

Using a similar design to the previous work, this study evaluates heterogeneous scar area in predicting appropriate ICD therapy for primary prevention of sudden cardiac death. Scar size was determined using thresholds of 4 and 6 standard deviations (SD) above the normal myocardium. Three different criteria were used to define heterogeneous scar area: the region between 2SD and 4SD (HSA_2-4SD_), between 2SD and 6SD (HSA_2-6SD_), and between 4SD and 6SD (HSA_4-6SD_). The study showed that scar size was similar in ICD therapy and non-ICD therapy groups. However, HSA_2-4SD_ and HSA_4-6SD_ were significantly larger in the ICD therapy group. On multivariable analysis, HSA_2-4SD_ was the only independent predictor of ICD therapy. If confirmed in larger series, heterogeneous scar area may be used for risk stratification of sudden cardiac death [136].

### Multi-contrast late enhancement CMR determined gray zone and papillary muscle involvement predict appropriate ICD therapy in patients with ischemic heart disease

Myocardial infarct heterogeneity was associated with appropriate ICD therapy in this cohort of patients with ischemic heart disease. Unlike the previous report, the gray zone area was defined by a novel multi-contrast late enhancement technique, which was more sensitive to predict appropriate ICD therapy when compared to the standard inversion recovery gradient echo sequence. Only the proportion of peri-infarct gray zone and papillary muscle infarct scores were significantly associated with appropriate ICD therapy for ventricular arrhythmias [137].

### The effect of microvascular obstruction and intramyocardial hemorrhage on contractile recovery in reperfused myocardial infarction: insights from cardiovascular magnetic resonance

The authors investigated the role of microvascular obstruction (MO) and intramyocardial hemorrhage (IMH) in the recovery of contractility of the infarcted myocardium. Overall, infarct zone strain recovered with time. By day 90, infarcts with MO had more attenuated strain in all myocardial layers compared to infarcts without MO; those with IMH were attenuated further. Infarct transmurality did not correlate with strain. On multivariable logistic regression, MO and IMH were the only independent predictors of attenuated 90-day infarct zone strain [138]. Reduced recovery of contractility may be a harbinger of adverse left ventricular remodeling and prognosis associated with MO and IMH, irrespective of infarct size.

### Inter-observer agreement and diagnostic accuracy of myocardial perfusion reserve quantification by cardiovascular magnetic resonance at 3 Tesla in comparison to quantitative coronary angiography

Myocardial perfusion reserve by CMR has shown to correlate with invasive evaluation of coronary artery disease and to yield good interobserver agreement at 1.5 Tesla. The aim of this study was to evaluate these parameters at 3 Tesla. Interobserver agreement was high for all myocardial perfusion territories. The diagnostic accuracy compared to coronary angiography was good for the RCA and LAD territories, but only moderate for the LCX territory [139]. This difference in accuracy may be explained by the lower signal-to-noise ratio in the thinner lateral wall and distance to the surface coils.

### Relationship between coronary flow reserve evaluated by phase-contrast cine cardiovascular magnetic resonance and serum eicosapentaenoic acid

Long-term intake of long-chain n-3 polyunsaturated fatty acids (PUFAs), especially eicosapentaenoic acid (EPA), is associated with a low risk for cardiovascular disease. This study investigated the relationship of coronary flow reserve (CFR) and serum EPA in patients with coronary artery disease. Breath-hold phase-contrast cine CMR images of the coronary sinus (CS) were acquired to assess blood flow of the CS both at rest and during adenosine infusion. Serum EPA positively correlated with CFR. Multivariate analysis revealed EPA to be an independent predictor of preserved CFR [140]. These findings suggest that improved endothelial function might be an important mechanism through which n-3 PUFAs prevent cardiovascular events.

### Variability and homogeneity of cardiovascular magnetic resonance myocardial T2-mapping in volunteers compared to patients with edema

T2-mapping is a promising tool for detecting and quantifying myocardial edema. This study evaluated the reproducibility and variability of myocardial T2 mapping in healthy volunteers and in patients with acute myocardial injury. Mapping revealed increased T2 in all patients with edema, and showed high intraobserver and interobserver variability, suggesting that this technique is both feasible and reproducible. However, in the volunteer group, global myocardial T2 differed slightly depending on the sequence and image orientation used, the latter likely due to partial volume and residual motion [141].

### Highly automatic quantification of myocardial oedema in patients with acute myocardial infarction using bright blood T2-weighted CMR

Some of the major challenges facing myocardial T2 quantification include manual delineation of the left ventricular boundaries and segmentation of myocardial edema. The authors propose a highly automatic framework for quantifying myocardial edema from bright blood T2-weighted CMR. The new method was tested in patients with acute myocardial infarction. The results showed accurate delineation of the edematous areas compared to manual quantification. Although the framework appears straightforward, future validation is needed in animal experiments and in larger clinical cohorts [142].

### Intra-thoracic fat volume is associated with myocardial infarction in patients with metabolic syndrome

Visceral adiposity is increased in patients with Metabolic Syndrome (MetS). The authors evaluated the associations between intra-thoracic fat volume (ITFV) and myocardial infarction in 94 subjects with MetS [143]. They found that ITFV was elevated in patients with MetS and was further increased in those with evidence of prior ischemic myocardial injury, as assessed by subendocardial LGE. They propose ITFV as a valuable marker of myocardial infarction risk in MetS.

### At-risk but viable myocardium in a large animal model of non ST-segment elevation acute coronary syndrome: cardiovascular magnetic resonance with ex vivo validation

Patients with NSTEMI have variable degrees of salvageable myocardium. The authors used a canine model of partial coronary stenosis combined with pacing to induce a reversible degree of ischemia [144]. Myocardial T2 was assessed as an indicator of at-risk myocardium. Ischemic, but not irreversibly injured (no LGE), myocardium showed significantly higher T2 than remote myocardium. T2-CMR of salvageable myocardium is an attractive biomarker for preclinical and clinical studies of NSTEMI.

## Gadolinium use in CMR

The additive information that can be gained in CMR by the administration of gadolinium based contrast agents is substantial, and these are being applied in new situations. There is now general awareness of the rare but potentially serious complication of nephrogenic systemic fibrosis (NSF) observed following administration of some earlier gadolinium agents, notably in the presence of significant renal impairment [145]. This had led to guidelines regarding the use of these agents and surveys of their use [146,147].

### Effectiveness of late gadolinium enhancement to improve outcomes prediction in patients referred for cardiovascular magnetic resonance after echocardiography

The authors studied whether CMR improves outcomes prediction after contemporaneous echocardiography in a prospective study of 444 patients clinically referred for CMR [148]. The ejection fraction better predicted mortality (30 deaths at median 0.8 years) than echo ejection fraction. Furthermore, CMR with LGE further improved risk stratification for death beyond ejection fraction and predicted transplant free survival after adjustment for age, gender, wall motion and ejection fraction. This paper demonstrates the incremental added prognostic value of CMR in patients referred for CMR in addition to echocardiography.

### Evaluation of current algorithms for segmentation of scar tissue from late Gadolinium enhancement cardiovascular magnetic resonance of the left atrium: an open-access grand challenge

CMR continues to play an increasingly important role for quantifying LA fibrosis and scar before and after ablation procedures for AF. A standardised evaluation benchmarking framework is presented for algorithms segmenting fibrosis and scar from LGE CMR images [149]. The aim is to guide treatment stratification of patients with atrial fibrillation (AF) and for assessment of treatment after radio frequency catheter ablation (RFCA). The framework enables comparison of scar segmentation algorithms in the LA for pre- and post-ablation fibrosis and scar. It compares eight algorithms that previously have only been tested on centre- and vendor-specific images. The proposed framework evaluated 8 different algorithms and measured their performance on a common scale. Reference standards for evaluation were established. Following evaluation, no algorithm was deemed clearly better than the others. This study highlights the opportunity for future development of algorithms however robust benchmarking is important.

## Myocardial mechanics

Measuring cardiac function is a fundamental for CMR. Myocardial tagging remains a source of research, [150] and at 3T has attracted interest, [151,152] partly because of the improved tag persistence with longer T1. Newer techniques have also been reported in humans, [153] including accelerated 3D techniques [154]. Feature tracking has become an area of active interest with development of simple to use software that can be used post-hoc on simple cines, [155,156] however the lack of validation at segmental level remains an issue. The papers below pursue novel aspects of cardiac function.

### Global and regional left ventricular myocardial deformation measures by magnetic resonance feature tracking in healthy volunteers: comparison with tagging and relevance of gender

Feature tracking post processing of SSFP cines of 145 healthy volunteers was used to record LV deformation parameters [157]. In 20 volunteers, values were also obtained by CMR tagging for comparison of global but not segmental measurements. For the globally averaged measurements of strain, only those measured circumferentially in short axis slices showed reasonably good levels of agreement between FT and tagging (limits of agreement −0.06 to 0.04). Longitudinal strain showed wide limits of agreement (−0.16 to 0.03) with evidence of overestimation of strain by FT relative to tagging as the mean of both measures increased. Radial strain was found to be systematically overestimated by FT relative to tagging with relatively wide limits of agreement. Provisional ranges of FT deformation parameters at global, regional and segmental levels were reported, showing evidence of variation with gender and myocardial region in the volunteers studied, although the segmental values had not been validated by comparison with measurements by tagging.

### Longitudinal strain from velocity encoded cardiovascular magnetic resonance: a validation study

Heiberg and colleagues measured regional wall strain from long axis turbo field echo (TFE) velocity encoded CMR in three long axis planes in 36 healthy volunteers and 10 patients with recent myocardial infarction [158]. Fast field echo (FFE) cines were acquired in the same planes to help delineate myocardial borders. A phantom experiment was performed to use optical tracking of elastic deformation as an independent gold standard for comparison. Excellent agreement was found between longitudinal strain measured by optical tracking and longitudinal strain measured with TFE velocity encoding. Mean longitudinal strain in patients was less than that in volunteers, and strain in infarcted regions, less than in remote areas. The authors concluded that the technique could quantify longitudinal strain and regional myocardial wall function and report normal values by the method.

### Inter-study reproducibility of cardiovascular magnetic resonance tagging

The test-retest reliability of the measurement of regional myocardial function by CMR tagging by spatial modulation of magnetization, analysed using harmonic phase (HARP), was investigated in 25 participants [159]. The role of altered slice orientation on strain measurements was investigated. The intraobserver and interobserver reproducibility of all strain and torsion parameters measured was reported to be excellent. Variations of endocardial circumferential strain due to altered slice orientation were found to be negligible compared to those doe to different slice location.

### Efficient and reproducible high resolution spiral myocardial phase velocity mapping of the entire cardiac cycle

This study demonstrates the feasibility of a novel myocardial velocity acquisition technique [160]. The image acquisition method described uses a spiral k space coverage to provide high temporal and spatial resolution cardiac motion images that further our general knowledge about myocardial motion mechanics. The method also, for the first time, uses retrospective cardiac gating which enables the atrial systolic motion to be measured. Good reproducibility of scans in the same subjects is also reported. The authors concluded that their retrospectively gated spiral phase velocity mapping sequence is an efficient and reproducible method of acquiring 3-directional, high resolution velocity data throughout the entire cardiac cycle, including atrial systole.

### Feature tracking measurement of dyssynchrony from cardiovascular magnetic resonance cine acquisitions: comparison with echocardiographic speckle tracking

This paper reports quantification of LV dyssynchrony by feature tracking post processing of routine CMR cine acquisitions (FT-CMR) in comparison to speckle tracking echocardiography in 72 consecutive patients who also had echocardiography, analysed by speckle tracking [161]. The CMR method delivered measurements of radial dyssynchrony which, at least for the patients with more marked dyssynchrony, showed reasonable agreement with those from speckle tracking echocardiography. The clinical usefulness of the method, for example in predicting prognosis in CRT patients, remains to be investigated.

### Relationship of phasic left atrial volume and emptying function to left ventricular filling pressure: a cardiovascular magnetic resonance study

In this study, CMR was performed in 41 patients on the same day as clinically indicated left heart catheterization [162]. This allowed left atrial volume (LAV) and emptying fraction (LAEF), measured by the biplane area and length method, to be related to left ventricular end diastolic pressure (LVEDP). Average LV ejection fraction was 49 ± 16% ranging from 10% to 74% and LVEDP by catheterization 14 ± 8 mmHg ranging from 4 mmHg to 32 mmHg. Of those analysed, increased LAVmin and decreased LAEFTotal were found to the most effective identifiers of LVEDP.

### Novel insight into the detailed myocardial motion and deformation of the rodent heart using high-resolution phase contrast cardiovascular magnetic resonance

Phase contrast velocimetry cardiovascular magnetic resonance (PC-CMR) is a powerful tool for assessment of in vivo motion of the myocardium. In this study, the authors demonstrate in the rat heart that PC-CMR can measure in vivo circumferential strain in addition to myocardial motion [163]. This work provides a refined tool for longitudinal assessment of regional function in rodents with a high level of detail.

### Reproducibility of cine displacement encoding with stimulated echoes (DENSE) cardiovascular magnetic resonance for measuring left ventricular strains, torsion, and synchrony in mice

Advanced measures of cardiac function, beyond LV volumes and ejection fraction, are increasingly important due to their superior diagnostic and predictive capabilities. Cine DENSE CMR is a powerful method in this regard, and the authors examined its reproducibility in mice [164]. They found measures of strains, torsion and synchrony to be highly reproducible. However, myocardial twist angles were not reproducible, and they recommend that future studies should instead report torsion.

### Obesity reduces left ventricular strains, torsion, and synchrony in mouse models: a cine displacement encoding with stimulated echoes (DENSE) cardiovascular magnetic resonance study

Obesity is now common, and it increases cardiovascular mortality; however, the effect of obesity on cardiac function in animal models is not well-defined. Studying mice randomized to a high-fat or low-fat diet at 7 T using a cine DENSE protocol, the authors found an increase in left ventricular mass by 15%, a 40% reduction in subepicardial circumferential strain, a 53% reduction in radial strain, a 34% decrease in peak torsion, as well as significant dyssynchrony in obese mice [165]. This model can serve to investigate strategies to protect the heart in obesity.

## Ventricular & Atrial volumes and motion

Given increasing availability and uptake of CMR, it is ever more important to determine the appropriate application of CMR derived values to clinical use. Although there is good correlation between ejection fraction measured by CMR and echocardiography, the values are not interchangeable. Consequently, where an evidence base has been established in one modality, the threshold value cannot necessarily accurately and reliably be transferred across modalities [166]. CMR volumes can guide prognosis [167]. Newer techniques still not included in clinical practice include myocardial tissue phase mapping [168,169].

### Atlas-based analysis of cardiac shape and function: correction of regional shape bias due to imaging protocol for population studies

The Cardiac Atlas Project is a worldwide consortium seeking to pool cardiac imaging data in a standardized manner from multiple studies to facilitate meta-analyses [170]. Atlas-based analysis allows quantification of shape and motion differences between disease groups and normal subjects. However, apparent shape differences may arise from differences of imaging protocol between studies. This study used a mathematical model describing regional wall motion and ventricular shape to establish a coordinate system registered to the cardiac anatomy [171]. The model was applied to data from studies using either steady state free precession or gradient recalled echo CMR. Shape bias attributable to the acquisition method was effectively removed using the atlas-based transformation, generated from a set of 46 volunteers who had been imaged by both methods. This allowed direct comparison of regional wall motion abnormalities between differently imaged cohorts.

### Evaluation of left ventricular diastolic function by fractional area change using cine cardiovascular magnetic resonance: a feasibility study

This study of LV diastolic function was based on CMR fractional area change (FAC) measured from mid short axis cines in 59 patients compared with echocardiographic transmitral and tissue Doppler indices [172]. The CMR diastolic index (%FAC during the first 30% of diastole) decreased with worsening diastolic dysfunction. It was lower (p < 0.0001) in patients classed by echo as having impaired relaxation (32.4 ± 7.5), pseudonormal filling (25.4 ± 5.6) and restrictive filling (9.5 ± 1.5), compared to those with normal diastolic function (67.7 ± 10.8). It correlated positively with early diastolic tissue Doppler mitral annular velocity (r = 0.75, p < 0.0001).

### Cardiac steatosis and left ventricular function in men with metabolic syndrome

Accumulation of fat around visceral organs may have detrimental effects; lipid oversupply to cardiomyocytes may lead to lipotoxicity, which has been associated with impaired left ventricular function. The authors studied men with metabolic syndrome and those without, using CMR and ^1^H-MRS. They found greater fat deposits in metabolic syndrome, and the amount of epicardial and pericardial fat correlated inversely with LV diastolic function; however, myocardial triglyceride content did not [173]. This study shows that the relationships amongst fat depots, cardiomyocyte fat accumulation and LV function are complex and merit further study.

### Reference right atrial dimensions and volume estimation by steady state free precession cardiovascular magnetic resonance

CMR is the standard for estimating the size of the cardiac chambers. This study defines normal ranges for right atrial dimensions and volumes adjusted to age, gender, and body surface area. It also provides the best predictors of right atrial enlargement from two-dimensional measurements [174].

### ECG-based gating in ultra high field cardiovascular magnetic resonance using an independent component analysis approach

This study investigated a particular problem for CMR in the small but growing number of centres investigating the applicability of operating at 7T [175]. The manuscript described a novel approach to ECG triggering using a 12-lead ECG system with Independent Component Analysis (ICA). This is a real problem where a good solution would be worth finding and the authors demonstrated that their ICA method outperformed the state-of-the-art VCG-based technique in this challenging environment.

### High spatial and temporal resolution retrospective cine cardiovascular magnetic resonance from shortened free breathing real-time acquisitions

This manuscript describes the development of a method for high quality cine imaging of the heart during free breathing [176]. The approach used a novel retrospective reconstruction scheme which was shown to shorten the required acquisition. The reconstruction algorithm employs non-rigid registration to remove respiratory motion and SPIRiT non-linear reconstruction with temporal regularization to fill in missing data. The resulting cine loops have high spatial and temporal resolution and perform well for volumetric measurement when compared to conventional breath-hold approaches.

### Fast and fully automatic calibration of frequency offset for balanced steady-state free precession cardiovascular magnetic resonance at 3.0 Tesla

This paper describes a novel automatic frequency scouting procedure designed for balanced SSFP imaging, especially at higher field strengths [177]. The method uses a low flip angle balanced SSFP scan to generate images at various frequency offsets, and then uses an automated algorithm to find the approximate optimal frequency. The method was assessed with various breath-hold and non-breath-hold approaches to recommend the best protocol to use in practice. The authors conclude that this fully automatic method could greatly reduce dark-band artifacts in bSSFP images and facilitate clinical cardiac MR routines at 3T.

### Cardiac-respiratory self-gated cine ultra-short echo time (UTE) cardiovascular magnetic resonance for assessment of functional cardiac parameters at high magnetic fields

This paper performs a careful evaluation on an Ultra-short TE (UTE) self-navigated approach to cardiac imaging on a 9.4Tesla mouse scanner [178]. The authors propose that the UTE sequence overcomes flow and electrocardiogram-trigger artifacts. The results demonstrate that image artefacts are reduced with this method, but SNR is also. By all presented metrics the UTE approach appeared to be more robust. The authors conclude the method to be a powerful alternative for the assessment of cardiac function at high magnetic fields.

### Evaluation of a subject specific dual-transmit approach for improving B1 field homogeneity in cardiovascular magnetic resonance at 3T

Dual source parallel transmit has now been implemented on a large number of CMR scanners worldwide. This manuscript describes a study to investigate B1 shimming on a parallel transmit system for cardiac cine imaging [179]. *In vivo* shimming results are compared with body type, as characterized by BMI, BSA, and AP/RL. Results suggested that in the absence of RF shimming, local B_1_ field homogeneity does not depend on body type. However, cardiac B_1_ field homogeneity can be significantly improved by performing local RF shimming with 2 independent RF-transmit channels. The results therefore indicated the need for subject-specific RF shimming. Further work is required to establish the impact of these methods.

### Real-time cardiovascular magnetic resonance at 1.5 T using balanced SSFP and 40 ms resolution

This manuscript describes the implementation and early clinical testing of a radial balanced SSFP acquisition with a regularized image reconstruction at 1.5T [180]. The study describes interesting, original and potentially important methods as it demonstrates ungated acquisitions acquired in an ultrasound fashion to overcome gating issues, observe beat-to-beat variations, and reduce scan time. Abnormalities in the cardiac cycle such as extra-systolic contractions are clearly visible in 1D projections. At present the reconstruction times are limiting for true real-time but in the future the methods are likely to be important for truly interactive scanning.

## Varia

There are always papers which do not fall into simple categories. This section pulls together such varia which has included reviews, [181-183] safety, [184,185] society and registry reports, [186] and novel, or unusual techniques.

### Moderate intensity supine exercise causes decreased cardiac volumes and increased outer volume variations: a cardiovascular magnetic resonance study

The effects on left and right ventricular (LV, RV) volumes during physical exercise remain controversial. Furthermore, no previous study has investigated the effects of exercise on longitudinal contribution to stroke volume (SV) and the outer volume variation of the heart. The aim of this study was to determine if LV, RV and total heart volumes (THV) as well as cardiac pumping mechanisms change during physical exercise compared to rest using CMR [187]. 26 healthy volunteers (6 women) underwent CMR at rest and exercise. Exercise was performed using a custom built ergometer for one-legged exercise in the supine position during breath hold imaging. Cardiac volumes and atrio-ventricular plane displacement were determined. Heart rate (HR) was obtained from ECG. HR increased during exercise from 60±2 to 94±2 bpm, (p<0.001). LVEDV remained unchanged (p=0.81) and LVESV decreased with −9±18% (p<0.05) causing LVSV to increase with 8±3% (p<0.05). RVEDV and RVESV decreased by −7±10% and −24±14% respectively, (p<0.001) and RVSV increased 5±17% during exercise although not statistically significant (p=0.18). Longitudinal contribution to RVSV decreased during exercise by −6±15% (p<0.05) but was unchanged for LVSV (p=0.74). THV decreased during exercise by −4±1%, (p<0.01) and total heart volume variation (THVV) increased during exercise from 5.9±0.5% to 9.7±0.6% (p<0.001). The authors concluded that cardiac volumes and function are significantly altered during supine physical exercise. THV becomes significantly smaller due to decreases in RVEDV whilst LVEDV remains unchanged. THVV and consequently radial pumping increases during exercise which may improve diastolic suction during the rapid filling phase

### Standardized cardiovascular magnetic resonance (CMR) protocols 2013 update.

This document is an update to the 2008 publication of the Society for Cardiovascular Magnetic Resonance (SCMR) Board of Trustees Task Force on Standardized Protocols [188]. Since the time of the original publication, 3 additional task forces (Reporting, Post-Processing, and Congenital Heart Disease) have published documents that should be referred to in conjunction with the present document. The section on general principles and techniques has been expanded as more of the techniques common to CMR have been standardized. There is still a great deal of development in the area of tissue characterization/mapping, so these protocols have been in general left as optional. The authors hope that this document continues to standardize and simplify the patient-based approach to clinical CMR. It will be updated at regular intervals as the field of CMR advances.

### Impact of cardiovascular magnetic resonance on management and clinical decision-making in heart failure patients

CMR can provide important diagnostic and prognostic information in patients with heart failure. However, in the current health care environment, use of a new imaging modality like CMR requires evidence for direct additive impact on clinical management. The authors evaluated the impact of CMR on clinical management and diagnosis in patients with heart failure [189]. 150 consecutive patients with heart failure and an ejection fraction ≤ 50% referred for CMR were prospectively studied. Definitions for "significant clinical impact" of CMR were pre-defined and collected directly from medical records and/or from patients. Categories of significant clinical impact included: new diagnosis, medication change, hospital admission/discharge, as well as performance or avoidance of invasive procedures (angiography, revascularization, device therapy or biopsy). Overall, CMR had a significant clinical impact in 65% of patients. This included an entirely new diagnosis in 30% of cases and a change in management in 52%. CMR results directly led to angiography in 9% and to the performance of percutaneous coronary intervention in 7%. In a multivariable model that included clinical and imaging parameters, presence of late gadolinium enhancement (LGE) was the only independent predictor of "significant clinical impact" (OR 6.72, 95% CI 2.56-17.60, p=0.0001). The authors concluded that CMR made a significant additive clinical impact on management, decision-making and diagnosis in 65% of heart failure patients. This additive impact was seen despite universal use of prior echocardiography in this patient group. The presence of LGE was the best independent predictor of significant clinical impact following CMR. An erratum was also published [190].

### Highlights of the 16th annual scientific sessions of the society for cardiovascular magnetic resonance

The 16th Annual Scientific Sessions of the Society for Cardiovascular Magnetic Resonance (SCMR) took place in San Francisco, USA at the end of January 2013. With a faculty of experts from across the world, this congress provided a wealth of insight into cutting-edge research and technological development. This review article provides a highlight of what represented the most significant advances in the field of CMR during this year's meeting [191].

### Quality assessment of cardiovascular magnetic resonance in the setting of the European CMR registry: description and validation of standardized criteria

CMR has become an important diagnostic imaging modality in cardiovascular medicine. However, insufficient image quality may compromise its diagnostic accuracy. This paper describes and validates standardized criteria to evaluate a) cine steady-state free precession (SSFP), b) late gadolinium enhancement (LGE), and c) stress first-pass perfusion images [192]. These criteria will serve for quality assessment in the setting of the Euro-CMR registry. Thirty-five qualitative criteria were defined (scores 0–3) with lower scores indicating better image quality. In addition, quantitative parameters were measured yielding 2 additional quality criteria, i.e. signal-to-noise ratio (SNR) of non-infarcted myocardium (as a measure of correct signal nulling of healthy myocardium) for LGE and % signal increase during contrast medium first-pass for perfusion images. These qualitative and quantitative criteria were assessed in a total of 90 patients (60 patients scanned at our own institution at 1.5T (n=30) and 3T (n=30) and in 30 patients randomly chosen from the Euro-CMR registry examined at 1.5T). Analyses were performed by 2 SCMR level-3 experts, 1 trained study nurse, and 1 trained medical student. The global quality score was 6.7±4.6 (n=90, mean of 4 observers, maximum possible score 64), range 6.4-6.9 (p=0.76 between observers). It ranged from 4.0-4.3 for 1.5T (p=0.96 between observers), from 5.9-6.9 for 3T (p=0.33 between observers), and from 8.6-10.3 for the Euro-CMR cases (p=0.40 between observers). The inter- (n=4) and intra-observer (n=2) agreement for the global quality score, i.e. the percentage of assignments to the same quality tertile ranged from 80% to 88% and from 90% to 98%, respectively. The agreement for the quantitative assessment for LGE images (scores 0–2 for SNR <2, 2–5, >5, respectively) ranged from 78-84% for the entire population, and 70-93% at 1.5T, 64-88% at 3T, and 72-90% for the Euro-CMR cases. The agreement for perfusion images (scores 0–2 for %SI increase >200%, 100%-200%,<100%, respectively) ranged from 81-91% for the entire population, and 76-100% at 1.5T, 67-96% at 3T, and 62-90% for the Euro-CMR registry cases. The intra-class correlation coefficient for the global quality score was 0.83. The authors concluded that the criteria for the assessment of CMR image quality are robust with a good inter- and intra-observer agreement. Further research is needed to define the impact of image quality on the diagnostic and prognostic yield of CMR studies.

### Cost-effectiveness analysis for imaging techniques with a focus on cardiovascular magnetic resonance

With the need for healthcare cost-containment, increased scrutiny will be placed on new medical therapeutic or diagnostic technologies. Several challenges exist for a new diagnostic test to demonstrate cost-effectiveness. New diagnostic tests differ from therapeutic procedures due to the fact that diagnostic tests do not generally directly affect long-term patient outcomes. Instead, the results of diagnostic tests can influence management decisions for patients and by this route, diagnostic tests indirectly affect long-term outcomes. The benefits from a specific diagnostic technology depend therefore not only on its performance characteristics, but also on other factors such as prevalence of disease, and effectiveness of existing treatments for the disease of interest. We review the concepts and theories of cost-effectiveness analyses (CEA) as they apply to diagnostic tests in general. In this paper, the limitations of CEA across different study designs and geographic regions are discussed, and the strengths and weakness of the existing publications are examined where CMR was the focus of CEA compared to other diagnostic options [193].

### Imaging in population science: cardiovascular magnetic resonance in 100,000 participants of UK Biobank - rationale, challenges and approaches

UK Biobank is a prospective cohort study with 500,000 participants aged 40 to 69. Recently an enhanced imaging study received funding. CMR will be part of a multi-organ, multi-modality imaging visit in 3–4 dedicated UK Biobank imaging centres that will acquire and store imaging data from 100,000 participants (subject to successful piloting). In each of UK Biobank's dedicated bespoke imaging centres, it is proposed that 15–20 participants will undergo a 2 to 3 hour visit per day, seven days a week over a period of 5–6 years. The imaging modalities will include brain MRI at 3 Tesla, CMR and abdominal MRI at 1.5T, carotid ultrasound and DEXA scans using carefully selected protocols. We reviewed the rationale, challenges and proposed approaches for concise phenotyping using CMR on such a large scale. Here, we discuss the benefits of this imaging study and review existing and planned population based cardiovascular imaging in prospective cohort studies. The study will evaluate the CMR protocol, feasibility, process optimisation and costs [194]. Procedures for incidental findings, quality control and data processing and analysis are also presented. As is the case for all other data in the UK Biobank resource, this database of images and related information will be made available through UK Biobank's Access Procedures to researchers (irrespective of their country of origin and whether they are academic or commercial) for health-related research that is in the public interest.

### Cardiovascular magnetic resonance artefacts

The multitude of applications offered by CMR make it an increasing popular modality to study the heart and the surrounding vessels. Nevertheless the anatomical complexity of the chest, together with cardiac and respiratory motion, and the fast flowing blood, present many challenges which can possibly translate into imaging artefacts. The literature is wide in terms of papers describing specific MR artefacts in great technical detail. This review attempts to summarise, in a language accessible to a clinical readership, some of the most common artefacts found in CMR applications [195]. It begins with an introduction of the most common pulse sequences, and imaging techniques, followed by a brief section on typical cardiovascular applications. This leads to the main section on common CMR artefacts with examples, a short description of the mechanisms behind them, and possible solutions.

### Standardized image interpretation and post processing in cardiovascular magnetic resonance: Society for Cardiovascular Magnetic Resonance (SCMR) board of trustees task force on standardized post processing

With mounting data on its accuracy and prognostic value, CMR is becoming an increasingly important diagnostic tool with growing utility in clinical routine. Given its versatility and wide range of quantitative parameters, however, agreement on specific standards for the interpretation and post-processing of CMR studies is required to ensure consistent quality and reproducibility of CMR reports. This document addresses this need by providing consensus recommendations developed by the Task Force for Post Processing of the Society for Cardiovascular MR (SCMR) [196]. The aim of the task force is to recommend requirements and standards for image interpretation and post processing enabling qualitative and quantitative evaluation of CMR images. Furthermore, pitfalls of CMR image analysis are discussed where appropriate.

### European cardiovascular magnetic resonance (EuroCMR) registry – multi national results from 57 centers in 15 countries

This paper reports 27,000 patients consecutively enrolled in the multi-national EuroCMR registry, in which 34% of CMRs were indicated for risk stratification in suspected ischaemic heart disease, 32% for myocarditis or cardiomyopathy, and 15% for viability [197]. In 98% CMR was diagnostic and it impacted patient management in 62. CMR was safe. Interim analysis underscores the prognostic value of routinely available clinical CMR; a normal stress CMR was associated with a 1% risk per year for adverse events, and a LGE negative CMR with 2.7% risk per year of adverse events in hypertrophic cardiomyopathy.

### Cardiovascular magnetic resonance with an MR compatible pacemaker

FDA guidelines for MRI-conditional pacemakers preclude placing the heart at the center of the magnet’s bore and consequently the acquisition of CMR studies. In this manuscript, the authors describe a CMR protocol for patients with a Revo pacemaker system while operating within FDA guidelines, and the first CMR case in a patient with a Revo MRI-conditional pacing system despite position constraints [198].

### Magnetic resonance of the heart in a muscular dystrophy patient with an MR conditional ICD: Assessment of safety, diagnostic value and technical limitations

This is the first presentation of a CMR study in a patient with the world’s first MR-conditional ICD. In this case, a major problem related to the presence of the MR conditional ICD was artifact caused by the device’s generator which hampered the image quality in all sequences performed. Considering previous studies, right chest implantation of the ICD could probably have helped in this setting and may be preferred in future ICD implantations [199].

### Clinical Implications of cardiac hyperpolarized magnetic resonance imaging

This is a review of the novel CMR technique of ^13^C-hyperpolarization, which can augment the MR sensitivity of the ^13^C nucleus by 4–5 orders of magnitude [200]. Metabolic tracers [1-13C] and [2-13C] pyruvate have allowed significant advances in the understanding of real-time myocardial metabolism in the normal and diseased heart in vivo, in diabetes, ischaemic heart disease, cardiac hypertrophy and heart failure. This review considers results from animal models of disease and discusses how these may translate into clinical practice.

### In vivo mouse cardiac hyperpolarized magnetic resonance spectroscopy

This is the first report of the ^13^C-hyperpolarisation technique described above in the mouse heart, while previous experimental studies have used larger animal species [201]. This is important due to the ability to manipulate specific genes in the mouse. The in vivo metabolism of [1-^13^C]pyruvate was investigated; overnight fasting and infusion of sodium dichloroacetate were used to detect alterations in pyruvate dehydrogenase (PDH) flux. A comparison of three commonly used control mouse strains was performed revealing significant metabolic differences between strains. Thus [1-^13^C]pyruvate can be used to provide an in vivo cardiac metabolic profile of transgenic mice.

### Cardiovascular magnetic resonance of total and atrial pericardial adipose tissue: a validation study and development of a 3 dimensional pericardial adipose tissue model

Pericardial adipose tissue (PAT) is an independent predictor of atrial fibrillation (AF), and atrial PAT may create a substrate for AF. Using CMR, the authors developed and validated a three-dimensional atrial PAT model in sheep [202]. CMR-derived PAT estimates corresponded to autopsy measurements, and reliability of CMR measures was high. They conclude that the measurement of local cardiac fat stores via this methodology could provide a sensitive tool to examine the regional effect of fat deposition on atrial substrate, which potentially may influence AF ablation strategies in obese patients.

### Assessment of the right ventricle with cardiovascular magnetic resonance at 7T

It would be desirable given the increasing emphasis on RV mass to predict outcomes to achieve higher spatial resolution for better RV free wall depiction. In 9 healthy volunteers CMR fast gradient echo cine (FGRE) imaging at 7T was performed for functional and morphological right ventricular assessment and compared with 1.5T steady state free precession (SSFP) cine imaging [203]. There was good agreement with ventricular volumes between 1.5T SSFP and 7T FGRE but FGRE at 7T (1.3×1.3×4mm voxels) tended to overestimate RV volume compared with SSFP at 1.5T (1.2×1.2×6mm voxels). FGRE cine imaging at 7T resulted in lower image homogeneity but received overall equal ratings for visually evaluated image contrast and quality.
